# Hearing Outcomes Following Ventriculoperitoneal Shunt Placement: A Scoping Review

**DOI:** 10.3390/jcm15114325

**Published:** 2026-06-03

**Authors:** Mukta Vibhute, Rachel Zhang, Anna Bareiss, Andrew R. Mangan, Kenny Nguyen, Emily Goodman, John Dornhoffer, Robert A. Saadi

**Affiliations:** 1 Department of Otolaryngology–Head and Neck Surgery, University of Arkansas for Medical Sciences, Little Rock, AR 72205, USA; mvibhute@uams.edu (M.V.); abareiss@uams.edu (A.B.); amangan@uams.edu (A.R.M.); egoodman@uams.edu (E.G.); jdornhoffer@uams.edu (J.D.); 2 College of Medicine, University of Arkansas for Medical Sciences, Little Rock, AR 72205, USA; rzhang@uams.edu

**Keywords:** ventriculoperitoneal shunt, hearing, hydrocephalus, cerebrospinal fluid pressure, scoping review, cochlear aqueduct, intracranial pressure

## Abstract

**Background/Objectives**: Ventriculoperitoneal shunt (VPS) placement alters cerebrospinal (CSF) dynamics and has been associated with hearing changes through pressure transmission via the cochlear aqueduct. Despite the large number of patients undergoing VPS placement annually, associated hearing changes remain poorly characterized. This scoping review aimed to characterize the available evidence on hearing outcomes following VPS placement and identify gaps warranting further investigation. **Methods**: This scoping review was conducted in accordance with PRISMA-ScR guidelines. PubMed was queried from January 1980 through January 2026. Eligible studies included case reports, case series, and observational cohorts reporting hearing outcomes following VPS placement. Two independent reviewers screened titles, abstracts, and full texts. Risk of bias was assessed using Joanna Briggs critical appraisal tools. Due to heterogeneity in study designs, outcomes metrics, and follow-up duration, quantitative synthesis was not performed. **Results**: Nineteen studies comprising approximately 200 patients met inclusion criteria. Hearing deterioration and improvement were each reported in 7 studies (36.8%); mixed outcomes were reported in 5 studies (26.3%). Acute hearing changes occurred within 48 h in 3 studies (15.8%), whereas delayed changes were described in 13 studies (68.4%). Overall quality of evidence was low. **Conclusions**: This scoping review identifies VPS placement as a clinically underrecognized contributor to hearing change. Current evidence is heterogenous and dominated by low level studies, underscoring the need for prospective investigation with standardized audiologic protocols. Preoperative audiograms should be considered when possible, as well as a low threshold for audiology referral for postoperative hearing concerns.

## 1. Introduction

Hydrocephalus is characterized by abnormal accumulation of cerebrospinal fluid (CSF) within the cerebral ventricles, most commonly due to obstruction of CSF flow or impaired absorption [1]. Progressive ventricular enlargement can lead to increased intracranial pressure (ICP), compression of adjacent brain structures, and neurological dysfunction [1]. Ventriculoperitoneal (VP) shunt placement is the most commonly performed surgical treatment for hydrocephalus, with approximately 30,000 procedures performed annually in the United States [2,3,4]. VP shunts function by diverting CSF from the ventricular to the peritoneal cavity, which relieves intracranial pressure. Although shunt placement can be lifesaving, it is also a lifelong intervention that requires ongoing surveillance. Shunt failure rates are significant, particularly in pediatric patients, and complications such as infection, obstruction, and failure create a need for revision procedures [2,5].

Changes in intracranial pressure may also affect structures outside the central nervous system. The cochlear aqueduct provides an anatomic connection between the subarachnoid space and the perilymphatic space of the inner ear, allowing CSF pressure changes to be transmitted to the cochlea [6]. The vestibular aqueduct and endolymphatic sac also aid in pressure regulation in the membranous labyrinth. Because of these connections, changes in intracranial pressure can affect inner ear fluid dynamics and cochlear function [7]. Ventriculoperitoneal shunts (VPS), especially in cases of over-drainage, may lead to intracranial hypotension and disrupt this delicate pressure equilibrium between the intracranial compartment and the inner ear, potentially producing hearing changes or vestibular symptoms [8].

The relationship between CSF dynamics and auditory function has been explored in several contexts. Transient hearing loss following lumbar puncture, spinal anesthesia, and other neurosurgical procedures involving CSF loss is well documented, with recovery typically occurring within days [6,9]. Satzer and Guillaume conducted a narrative review of the mechanisms linking hydrocephalus and hearing loss, identifying pressure transmission as the most plausible direct pathway [10]. However, that review focused on mechanistic pathways and specifically noted that studies comparing pre and post shunt hearing function were limited. The primary literature on this topic is largely comprised of case reports and small observational cohorts with considerable variability in onset, laterality, severity and reversibility. No prior scoping review has mapped these findings in aggregate.

Given the large number of patients who undergo VP shunting annually and the potential reversibility of shunt-related hearing changes with early recognition and valve adjustment, this association warrants broader clinical awareness. To the best of our knowledge, we conducted the first scoping review to map available evidence on hearing outcomes following ventriculoperitoneal shunt placement across pediatric and adult populations.

## 2. Materials and Methods

This scoping review was conducted in accordance with the Preferred Reporting Items for Systematic Reviews and Meta-Analyses extension for Scoping Reviews (PRISMA-ScR) [9]. This review was not prospectively registered. A medical librarian (L.B.) designed and executed a comprehensive search of PubMed from 1 January 1980, to 1 January 2026. The search strategy applied controlled vocabulary when available (i.e., Medical Subject Headings [MeSH]) and relevant keywords, including “ventriculoperitoneal shunt”, “hearing loss” and “hydrocephalus.” Animal studies, veterinary literature, and non-English publications were excluded; exclusion of non-English articles reflects absence of translation resources. A total of 244 citations were identified. After removal of two duplicates, 242 unique citations remained for title and abstract screening. Full search strategies are provided in Supplementary Item S1. A completed PRISMA 2020 checklist and PRISMA-ScR checklist are provided in Supplementary Tables S1 and S2.

Eligible studies included case reports, case series, retrospective cohort studies, prospective cohort studies, and cross-sectional studies that evaluated ventriculoperitoneal shunt (VPS) placement as an intervention and reported hearing outcomes. Both pediatric and adult populations were included. Studies were excluded if VPS was not evaluated as an intervention, if hearing loss etiologies were unrelated to hydrocephalus or CSF diversion, if original patient-level data were not reported, or if the publication was a conference abstract, editorial, commentary, non-English article, or animal study.

Two reviewers (M.V. and K.N.) independently screened titles and abstracts for eligibility. Full text review was performed for studies meeting inclusion criteria or when eligibility was unclear. Discrepancies were resolved via an independent third reviewer (R.Z). Of the 242 screened, 36 proceeded to full text review. Seventeen were excluded at full-text review: eight had no VPS intervention, five did not report hearing outcomes, three were reviews without original patient data, and one was a non-English article. Nineteen met final inclusion criteria after full text review.

Two reviewers (M.V. and R.Z.) independently extracted data from included studies using a standardized abstraction template. Extracted variables included study design, age, sex, indication for VPS placement, type of audiologic assessment, direction and timing of hearing change after VPS placement, magnitude of hearing change when reported, laterality, follow-up duration, and proposed physiologic mechanisms.

The primary outcome was change in hearing following VPS placement. Secondary outcomes included direction of hearing change (worsened, mixed, or improved), timing of hearing change (acute versus delayed), recovery of hearing, and proposed physiological mechanisms.

Hearing change timing was categorized as acute if it occurred within 48 h of VPS placement and delayed if it occurred after 48 h or during longer follow-up. Laterality was classified as unilateral or bilateral based on reported audiologic findings. Audiometric pattern was categorized according to study descriptions, most commonly sensorineural hearing loss (SNHL). Direction of hearing change was classified as worsened, improved, or mixed based on reported outcomes in each study. Hearing improvement was defined as any documented improvement in hearing thresholds, speech discrimination, or clinical hearing status after VPS placement. Hearing deterioration was classified as worsening audiometric thresholds or new hearing loss after VPS placement. Recovery was categorized as complete resolution, partial improvement, or persistent hearing loss based on audiologic findings during follow-up in each study.

Risk of bias was assessed according to the Joanna Briggs Institute (JBI) Critical Appraisal Checklist matched to study design, with separate checklists applied to case reports and cohort studies. Given the heterogeneity in study design, patient populations, outcome reporting, and follow-up duration across included studies, quantitative synthesis was not performed and findings were narratively summarized. This is consistent with the objectives of a scoping review, which aims to map breadth of available evidence rather than pool outcomes across studies. quantitative meta-analysis was not performed and findings were synthesized descriptively.

## 3. Results

### 3.1. Study Selection

**Figure 1:**
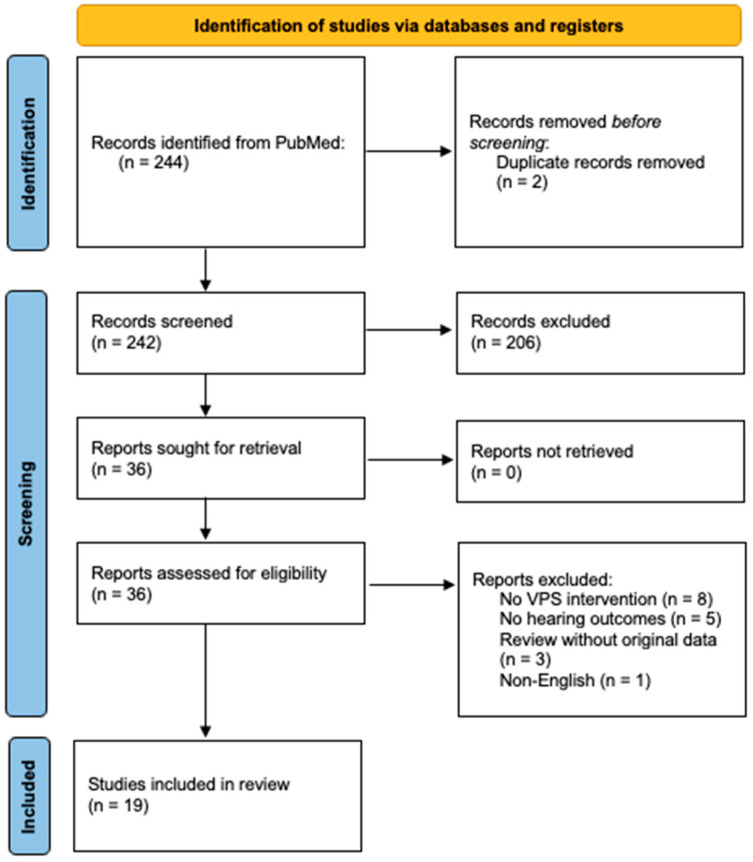
PRISMA flow diagram of study selection.

A total of 244 records were identified through database searching. After removal of 2 duplicates, 242 titles and abstracts were screened. Of the 36 studies assessed for full-text eligibility, 17 were excluded: 8 had no VPS intervention, 5 reported no hearing outcomes, 3 were reviews without original patient data, and 1 was a non-English article. Nineteen studies met inclusion criteria (Figure 1).

### 3.2. Study Characteristics

The 19 included studies comprised a total of 200 patients. Study designs included 12 case reports and 7 observational cohort or cross-sectional studies. No randomized or controlled studies were identified.

Both pediatric and adult populations were represented. Patient ages ranged from infancy to late adulthood. Sex distribution was variably reported and did not demonstrate a consistent predominance. Nine studies primarily involved pediatric patients, eight focused on adults, and two included mixed-age cohorts. Indications for VPS placement included normal pressure hydrocephalus (NPH), congenital hydrocephalus, intraventricular hemorrhage (IVH), spina bifida, and aqueductal stenosis, Chiari malformation, medulloblastoma-associated hydrocephalus, and communicating hydrocephalus.

Pure tone audiometry (PTA) was the primary hearing assessment modality across studies. Select studies additionally utilized electrocochleography, otoacoustic emissions, and auditory brainstem response testing. Study characteristics are summarized in Table 1.

### 3.3. Audiologic Findings

Across included studies, the most common audiometric pattern after VPS placement was sensorineural hearing loss. Pure tone audiometry was the primary diagnostic modality used, with select studies utilizing auditory brainstem response testing, otoacoustic emissions, or electrocochleography.

When reported, hearing changes were frequently unilateral and usually ipsilateral to the side of shunt placement. However, bilateral involvement was also commonly described. Audiometric patterns varied, including high-frequency SNHL, fluctuating hearing loss, and less commonly, auditory neuropathy.

### 3.4. Hearing Outcomes Following VPS Placement

**Figure 2:**
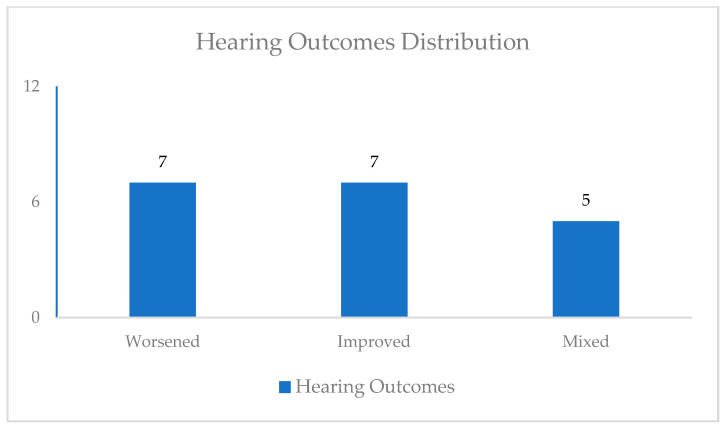
Distribution of hearing outcomes at the study level.

At the study level, hearing deterioration following VPS placement was described in 7 of 19 studies (36.8%), hearing improvement was reported in 7 studies (36.8%), typically in cases with elevated intracranial prior to shunting or following valve adjustment. Five studies (26.3%) reported mixed outcomes (Figure 2). Guillaume et al. reported hearing loss in 100% of shunted patients compared to 70% of non-shunted patients; however, all patients in this study received concurrent cisplatin chemotherapy and cranial radiation, which are independent risk factors for ototoxicity, limiting attribution of hearing loss to shunt placement alone.

Quantitative audiometric data were reported in a subset of studies (Table 2). Among these, both hearing decline and improvement were observed. One prospective cohort demonstrated a mean postoperative hearing decline of 11 ± 10 dB within the first week following shunt placement (*p* < 0.005), with partial recovery over time. In contrast, other studies reported modest improvements in pure tone average (PTA) following shunt placement or valve adjustment, including improvement of approximately 2–4 dB in the early postoperative time period, and larger improvements after valve pressure adjustment. Additionally, one study reported hearing improvement in 70% of patients with transient worsening in 30%, which resolved over time (*p* < 0.001).

### 3.5. Laterality and Audiometric Patterns

Unilateral hearing loss was reported in 5 studies (26.3%), and most commonly occurred ipsilateral to shunt placement. Bilateral hearing loss was also reported in 14 studies (73.7%).

In pediatric cohorts, unilateral high-frequency SNHL was commonly observed. Several studies suggested altered inner ear fluid dynamics as a potential mechanism in cases demonstrating ipsilateral deficits.

### 3.6. Timing and Reversibility of Hearing Changes

Onset of hearing changes ranged from immediate postoperative presentation within 48 h of shunt placement (3 studies, 15.8%) to delayed changes (13 studies, 68.4%). Three studies (15.8%) described mixed timing of hearing change (Table 3).

Recovery outcomes were variable, ranging from complete resolution to persistent hearing loss. Complete resolution was reported in 5 studies (26.3%), partial recovery in 4 studies (21.1%), no recovery in 5 studies (26.3%), and mixed recovery outcomes in 4 studies (21.1%). Valve pressure adjustment or shunt revision was associated with partial or complete improvement in several cases.

### 3.7. Quality of Evidence

The overall quality of evidence was limited by study design, with majority of included studies consisting of case reports and small observational cohorts. Case reports and case series generally demonstrated moderate risk of bias related to incomplete baseline audiologic data, inconsistent follow up, and limited assessment of alternate etiologies. Cohort studies showed moderate quality overall but were limited by small sample size, as well as variability in audiological protocols and outcome reporting. No randomized controlled studies were identified. Per-study risk of bias assessments are summarized in Table 4.

## 4. Discussion

### 4.1. Summary

This scoping review included 19 studies comprising of 200 patients and demonstrates that hearing changes following VPS placement vary in presentation and outcome. At the study level, hearing deterioration and improvement were each reported in 7 studies (36.8%), with mixed outcomes described in 5 studies (26.3%). Both pediatric and adult populations were affected. Recovery varied considerably and appears to be influenced by timing of recognition and shunt adjustment. These findings are hypothesis generating and should not be interpreted as estimates of population level incidence, given the predominance of case reports and small observational cohorts in the available literature.

### 4.2. Biological Plausibility and Mechanism

The observed audiologic changes are supported by established anatomic and physiologic connections between intracranial pressure (ICP) and inner ear fluid dynamics. The cochlear aqueduct provides a direct conduit for CSF-perilymph pressure transmission, while the vestibular aqueduct and endolymphatic sac contribute to pressure regulation within the membranous labyrinth [6,30]. VPS over-drainage may cause intracranial hypotension, leading to a relative perilymphatic pressure drop. The resulting pressure gradient may alter basilar membrane mechanics, promote endolymphatic hydrops-like changes, and impair outer hair cell function [7,8].

Acute cases may reflect rapid CSF pressure shifts, while chronic cases may represent long-term pressure disequilibrium or adaptive structural remodeling. Notably, several unilateral cases occurred ipsilateral to shunt placement, supporting the hypothesis of pressure gradient asymmetry rather than diffuse neural injury. Overall, these findings support a pressure mediated mechanism rather than direct cochlear nerve injury.

### 4.3. Acute vs. Delayed Hearing Loss

In this scoping review, acute hearing loss within 24–48 h of shunt placement likely reflects abrupt CSF pressure changes [21,22]. In contrast, delayed or chronic hearing loss may be due to ongoing pressure dysregulation [26,28]. Several studies in this review proposed CSF over-drainage as a primary mechanism for hearing deterioration after shunt placement [11,21,22]. Programmable valve adjustment or shunt revision was associated with hearing improvement in several cases, which suggests hearing loss in this setting may be dynamic and potentially reversible [11,24]. The reversibility observed in some cases further suggests a physiologic rather than purely structural etiology. Quantitative findings demonstrating early postoperative threshold shifts and recovery after valve adjustment further support a pressure-mediated and dynamic mechanism.

### 4.4. Hearing Improvement

Hearing improvement following VPS placement was reported in six studies, typically in patients with elevated intracranial pressure prior to shunting [15,16,25]. Quantitative findings demonstrated modest improvements in pure tone averages and more substantial improvements following valve adjustment. This suggests elevated ICP can impair cochlear mechanics, and that normalization of pressure may restore auditory mechanics. This bidirectional ICP-hearing relationship suggests that hearing changes may reflect changes in CSF pressure.

### 4.5. Pediatric vs. Adult Considerations

There are important differences between pediatric and adult populations. In children, the cochlear aqueduct is usually patent compared to adults, allowing for more direct transmission of intracranial pressure changes to the inner ear. Histologic studies of pediatric temporal bones have demonstrated the cochlear aqueduct is typically open early in life and becomes progressively narrower with age [31]. This anatomic difference may predispose pediatric patients to greater fluctuations in perlymphatic pressure after CSF diversion.

Additionally, pediatric patients often experience higher shunt revision rates and may have increased cranial compliance, potentially predisposing them to greater pressure fluctuations [26]. Several studies in this review reported high-frequency SNHL and fluctuating hearing changes in pediatric populations, which raises concern for cumulative physiologic effects over time. These findings suggest that children may be particularly vulnerable to VPS-related hearing changes and may benefit from routine audiological surveillance.

### 4.6. Clinical Implications

This scoping review highlights several clinical considerations. Baseline audiograms prior to VPS placement may help detect postoperative hearing changes. Many included studies lacked preoperative audiograms, limiting interpretation of true hearing threshold shifts. Post-operatively, new hearing complaints should prompt timely audiology referrals and evaluation for potential shunt over-drainage. Valve pressure adjustment may offer a noninvasive means of mitigating hearing changes and should be considered when clinically appropriate. Increased awareness of this association may allow earlier intervention and improve the likelihood of reversibility.

### 4.7. Strengths and Limitations

Strengths of this scoping review include being the first to systematically map hearing outcomes following VPS placement, comprehensive literature coverage from 1980 through 2026, inclusion of both pediatric and adult data, and structured abstraction of timing, laterality, and reversibility of hearing changes following VPS placement.

However, several limitations must be taken into account. This review was not prospectively registered with PROSPERO or another registry, which limits transparency and reproducibility. The literature search was limited to PubMed, and grey literature was not systematically searched. Non-English publications were excluded due to the absence of translation resources, which may introduce selection bias and limit completeness of the evidence base. The majority of included studies were case reports and small observational cohorts, which are susceptible to selection and reporting bias. Their inclusion was necessary given the limited available literature on this topic, but it constrains the strength of synthesis. One included study evaluated patients receiving concurrent cisplatin chemotherapy and cranial radiation, confounding the independent contribution of VPS placement to hearing outcomes. Quantitative meta-analysis was not feasible due to the degree of heterogeneity across included studies. Outcome measures were inconsistent, follow-up duration ranged from days to years, and patient populations differed substantially in age, hydrocephalus etiology, and shunt type. A formal GRADE assessment of evidence certainty was not performed due to the predominance of case reports and uncontrolled observational data. Overall certainty of evidence is expected to be low across all outcomes. Results are presented at the study level rather than the patient level. Study-level proportions do not reflect the distribution of outcomes across individual patients and should not be interpreted as estimates of population level incidence. Finally, the absence of preoperative audiologic baselines in many studies make it difficult to attribute postoperative hearing changes directly to VPS placement.

### 4.8. Future Directions

Prospective studies incorporating standardized audiological monitoring before and after VPS placement are needed to better define incidence and risk factors. Correlation of objective ICP measurements with audiometric thresholds may help clarify mechanistic pathways. Imaging studies evaluating cochlear aqueduct patency and inner ear fluid dynamics may further clarify factors contributing to the development of hearing changes. Development of standardized post-VPS audiologic surveillance protocols may aid in early detection and management.

## 5. Conclusions

This scoping review maps the available evidence on hearing outcomes following ventriculoperitoneal shunt placement and identifies this association as clinically underrecognized and incompletely characterized. Hearing changes following VPS placement may occur in either direction and vary in onset, laterality, and reversibility. Pediatric patients may be particularly susceptible due to greater cochlear aqueduct patency, which may allow more direct transmission of intracranial pressure changes to the inner ear. Current evidence is predominantly low-level and hypothesis generating. Preoperative audiologic assessment should be considered when feasible, and postoperative hearing complaints should prompt timely evaluation and audiology referral. Prospective studies with standardized audiologic protocols are needed to better define incidence, risk factors, and optimal surveillance strategies.

## Figures and Tables

**Table 1:** Characteristics of included studies.

Study	Year	Study Design	Population	N *	VPS Indication	Outcome Direction	Quantitative Data
Albanese et al. [11]	2007	Case report	Adult	1	Hydrocephalus	Improved	Y
Barlas et al. [12]	1983	Case report	Adult	1	Aqueductal Stenosis	Mixed	Y
Dolgun et al. [13]	2008	Case report	Adult	1	Chiari I Malformation	Improved	N
Guillaume et al. [14]	2012	Retrospective cohort	Pediatric	13	Medulloblastoma-associated hydrocephalus	Worsened	N
Jamshidi et al. [15]	2017	Case report	Pediatric	1	Hydrocephalus	Improved	N
Karabagli et al. [16]	2011	Case report	Adult	1	Elevated ICP	Improved	N
Lee et al. [17]	2007	Case report	Adult	1	NPH	Worsened	Y
Lim et al. [18]	2014	Prospective cohort	Adult	9	Hydrocephalus	Worsened	Y
Lopponen et al. [19]	1989	Cohort	Pediatric	47	Congenital hydrocephalus	Worsened	N
Lyle et al. [20]	2022	Case report	Pediatric	1	Hydrocephalus	Improved	N
Martinez-Perez et al. [21]	2018	Case report	Adult	1	Hydrocephalus	Mixed	N
Miyazaki et al. [22]	1997	Case report	Adult	1	Hydrocephalus	Worsened	N
Panova et al. [23]	2015	Observational study	Adult	70	Hydrocephalus	Mixed	N
Russell et al. [24]	2001	Case report	Adult	1	Hydrocephalus	Improved	N
Sammons et al. [25]	2009	Case report	Pediatric	1	Hydrocephalus	Improved	N
Spirakis et al. [26]	2003	Cross-sectional	Pediatric	12	Hydrocephalus	Worsened	N
Stoeckli et al. [27]	1999	Case report	Adult	1	Hydrocephalus	Worsened	N
Van Veelen-Vincent et al. [28]	2001	Prospective cohort	Adult	16	NPH	Mixed	Y
Verma et al. [29]	2018	Prospective cohort	Mixed	20	Hydrocephalus	Mixed	Y

* N represents the number of patients relevant to hearing outcome analysis for VPS-treated patients when reported.

**Table 2:** Quantitative Hearing Outcomes.

Study	N (Hearing Analysis)	Metric(s) Reported	Timing	Reported Change	*p*-Value
Albanese et al. [11]	1	PTA	Postoperative (after valve adjustment)	PTA: +18 dB improvement (b/L)	N/A
Barlas et al. [12]	1	PTA	Postoperative	PTA: +30 dB improvement (case 2)	N/A
Lee et al. [17]	1	PTA, SRT, WRS	Postoperative (after valve adjustment)	SRT 55 → 50 dB; WRS 72% → 84%	N/A
Lim et al. [18]	18 ears (T1), 14 ears (T2)	PTA	Postoperative (5 days, 1 month)	+2.7 ± 4.5 dB (T1); +4.2 ± 7.4 dB (T2)	*p* = 0.022 (T1); *p* = 0.037 (T2)
Van Veelen-Vincent et al. [28]	31 ears (16 pts)	Hearing level (dB)	Postoperative (1 week)	Hearing level: ↓ 11 ± 10 dB (max 30 dB); 64% worsened	*p* < 0.005
Verma et al. [29]	20 pts	PTA, BC thresholds	Postoperative	PTA: improvement in 70%, transient worsening in 30% (resolved)	*p* < 0.001
Martinez-Perez [21]	1	PTA, word discrimination	Postoperative (after shunt placement)	PTA: 85 db → 12.5 dB average decline; word discrimination 30% → 100%	N/A

PTA = pure tone average; SRT = speech recognition threshold; WRS = word recognition score; BC = bone conduction. N/A = not applicable. Positive dB values indicate threshold elevation (worsening of hearing). For Lim et al [18]., reported values of +2.7 ± 4.5 dB (T1) and +4.2 ± 7.4 dB (T2) represent mean hearing threshold increases following shunt placement, consistent with the “worsened” classification in Table 1.

**Table 3:** Audiologic findings and timing of hearing changes following ventriculoperitoneal shunt placement.

Study	Direction of Hearing Change	Timing of Hearing Change	Laterality	Audiometric Pattern	Recovery
Albanese et al. [11]	Improved	Delayed	Bilateral	SNHL	Yes
Barlas et al. [12]	Mixed	Delayed	Unilateral	Fluctuating SNHL	Partial
Dolgun et al. [13]	Improved	Delayed	Bilateral	SNHL	Partial
Guillaume et al. [14]	Worsened	Delayed	Bilateral	SNHL	No
Jamshidi et al. [15]	Improved	Delayed	Bilateral	SNHL	Yes
Karabagli et al. [16]	Improved	Delayed	Bilateral	SNHL	Partial
Lee et al. [17]	Worsened	Acute	Unilateral	SNHL	Partial
Lim et al. [18]	Worsened	Delayed	Bilateral	SNHL	No
Lopponen et al. [19]	Worsened	Delayed	Bilateral	High-frequency SNHL	No
Lyle et al. [20]	Improved	Delayed	Unilateral	Auditory neuropathy	Yes
Martinez-Perez et al. [21]	Mixed	Acute	Unilateral	Profound SNHL	Yes
Miyazaki et al. [22]	Worsened	Delayed	Bilateral	SNHL	Partial
Panova et al. [23]	Mixed	Mixed	Bilateral	SNHL	Mixed
Russell et al. [24]	Improved	Delayed	Bilateral	SNHL	Yes
Sammons et al. [25]	Improved	Delayed	Unilateral	SNHL	Yes
Spirakis et al. [26]	Worsened	Delayed	Unilateral	High-frequency SNHL	No
Stoeckli et al. [27]	Worsened	Acute	Bilateral	SNHL	No
Van Veelen-Vincent et al. [28]	Mixed	Mixed	Bilateral	SNHL	Mixed
Verma et al. [29]	Mixed	Mixed	Bilateral	SNHL	Mixed

SNHL = sensorineural hearing loss. Acute defined as ≤48 h postoperatively; delayed defined as >48 h or during follow-up. Recovery categorized as complete (yes), partial, or none based on study-reported outcomes.

**Table 4:** JBI Critical Appraisal Assessment. (**A**) Case Reports (JBI Case Report Checklist, 8 items). (**B**) Cohort and Observation Studies (JBI Cohort Study Checklist, 9 items).

(**A**)										
**Study**	**Q1**	**Q2**	**Q3**	**Q4**	**Q5**	**Q6**	**Q7**	**Q8**	**Risk of Bias**	
Albanese et al., 2007 [11]	Y	Y	Y	Y	Y	Y	N	Y	Low	
Barlas et al., 1983 [12]	Y	Y	Y	Y	Y	Y	N	Y	Low	
Dolgun et al., 2008 [13]	Y	Y	Y	Y	Y	Y	N	Y	Low	
Jamshidi et al., 2017 [15]	Y	Y	Y	Y	Y	Y	N	Y	Low	
Karabagli et al., 2011 [16]	Y	Y	Y	Y	Y	Y	N	Y	Low	
Lee et al., 2007 [17]	Y	Y	Y	Y	Y	Y	N	Y	Low	
Lyle et al., 2022 [20]	Y	Y	Y	Y	Y	Y	N	Y	Low	
Martinez-Perez et al., 2018 [21]	Y	Y	Y	Y	Y	Y	N	Y	Low	
Miyazaki et al., 1997 [22]	Y	Y	Y	Y	Y	Y	N	Y	Low	
Russell et al., 2001 [24]	Y	Y	Y	Y	Y	Y	N	Y	Low	
Stoeckli et al., 1999 [27]	Y	Y	Y	Y	Y	Y	N	Y	Low	
Sammons et al., 2009 [25]	Y	Y	Y	Y	Y	Y	N	Y	Low	
(**B**)										
**Study**	**Q1**	**Q2**	**Q3**	**Q4**	**Q5**	**Q6**	**Q7**	**Q8**	**Q9**	**Risk of Bias**
Guillaume et al., 2012 [14]	Y	Y	N	N	Y	Y	Y	Y	Y	Moderate
Lim et al., 2014 [18]	Y	Y	N	N	Y	Y	Y	Y	Y	Low
Lopponen et al., 1989 [19]	Y	Y	N	N	Y	U	Y	Y	Y	Moderate
Panova et al., 2015 [23]	Y	Y	N	N	Y	U	Y	Y	Y	Moderate
Spirakis et al., 2003 [26]	Y	Y	N	N	Y	N/A	N/A	Y	Y	Moderate
Van Veelen-Vincent et al., 2001 [28]	Y	Y	N	N	Y	N	Y	Y	Y	Low
Verma et al., 2018 [29]	Y	Y	N	N	Y	Y	Y	Y	Y	Low

A: Q1 = Patient demographics clearly described. Q2 = Patient history clearly described; Q3 = Clinical condition at presentation described; Q4 = Diagnostic tests/methods clearly described; Q5 = Intervention clearly described; Q6 = Post-intervention clinical condition described; Q7 = Adverse events identified; Q8 = Takeaway messages appropriate. B: Q1 = Inclusion criteria clearly defined; Q2 = Exposure measured validly and reliably; Q3 = Confounding factors identified; Q4 = Strategies to address confounders described; Q5 = Outcomes measured validly and reliably; Q6 = Follow-up complete; Q8 = Outcomes measured consistently; Q9 = Statistical analysis appropriate. A and B: Y = Yes; N = No; U = Unclear; N/A = Not applicable. Risk of bias reflects overall appraisal judgement. Case reports are inherently susceptible to selection and reporting bias; low risk ratings reflect internal reporting quality, not study design strength. Cohort studies consistently lacked identification or adjustment for confounding factors (Q3, Q4), reflecting the observation and often retrospective nature of the evidence base.

## Data Availability

No new data were generated or analyzed in support of this research.

## Supplementary Materials

The following supporting information can be downloaded at: https://www.mdpi.com/article/10.3390/jcm15114325/s1, Figure S1: PubMed search strategies. Table S1: PRISMA 2020 CHECKLIST [32]; Table S2: Preferred Reporting Items for Systematic reviews and Meta-Analyses extension for Scoping Reviews (PRISMA-ScR) Checklist.

## Abbreviations

The following abbreviations are used in this manuscript:

CSF	Cerebrospinal fluid
ICP	Intracranial pressure
JBI	Joanna Briggs Institute
NPH	Normal pressure hydrocephalus
PRISMA-ScR	Preferred Reporting Items for Systematic Reviews and Meta-Analyses extension for Scoping Reviews
PTA	Pure tone average
SNHL	Sensorineural hearing loss
VPS	Ventriculoperitoneal shunt
