# Supplementary figures and images for: Hearing Outcomes Following Ventriculoperitoneal Shunt Placement: A Scoping Review
Source: J Clin Med. 2026 Jun 3;15(11):4325. doi: 10.3390/jcm15114325 (PMC13257779; doi:10.3390/jcm15114325)

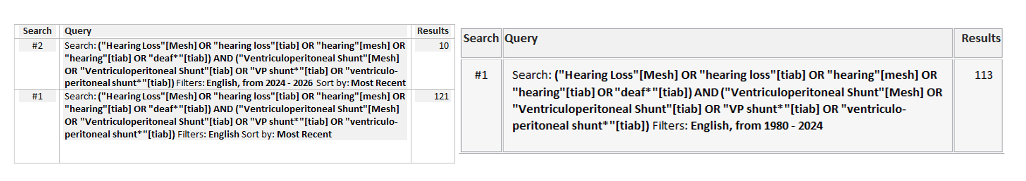

Supplement: Supplementary file 1 [file jcm-15-04325-s001.zip › S1_VPS.png]
